# Proposal for an Expanded “R” Classification: Impact of Positive Surgical Margin Length on Biochemical Recurrence After Robotic Radical Prostatectomy

**DOI:** 10.3390/jcm14124310

**Published:** 2025-06-17

**Authors:** Alper Kerem Aksoy, Ahmet Tahra, Resul Sobay, Ali Kumcu, İlkay Tosun, Uğur Boylu, Eyüp Veli Küçük

**Affiliations:** 1 Department of Urology, Ümraniye Training and Research Hospital, Ümraniye, İstanbul 34764, Turkey; ahmet.tahra@sbu.edu.tr (A.T.); resul.sobay@sbu.edu.tr (R.S.); ali.kumcu@sbu.edu.tr (A.K.); ugur.boylu@sbu.edu.tr (U.B.); eyupveli.kucuk@sbu.edu.tr (E.V.K.); 2 Department of Pathology, Ümraniye Training and Research Hospital, Ümraniye, İstanbul 34764, Turkey; ilkay.tosun@sbu.edu.tr

**Keywords:** biochemical recurrence, positive surgical margin, prostate cancer, radical prostatectomy, prognostic factors, TNM classification

## Abstract

**Objectives**: In this study, the effect of positive surgical margin (PSM) length on predicting postoperative biochemical recurrence (BCR) after radical prostatectomy was evaluated, and based on the findings, an additional R subclassification to the TNM-R system was proposed. **Methods**: We retrospectively analyzed patients who underwent robot-assisted radical prostatectomy between 30 July 2008 and 31 December 2019. Only patients with PSM were included. Those with negative margins, those receiving neoadjuvant/adjuvant hormone therapy, or those with prior pelvic radiotherapy were excluded. A total of 353 pathology specimens were re-evaluated by a uropathologist, and the PSM length was quantitatively measured. BCR was defined as a PSA level of ≥0.2 ng/mL in two consecutive measurements. **Results**: The median follow-up time of the patients was 49.5 ± 33.4 months. BCR occurred in 27.1% (n = 96) of patients. A PSM cut-off length of 3.5 mm was identified for predicting BCR (*p* < 0.001). Among patients with PSM < 3.5 mm, 9.8% experienced BCR, while 54.3% of those with PSM ≥ 3.5 mm did. A PSM length ≥ 3.5 mm was associated with a higher risk of recurrence (OR: 1.249, 95% CI: 1.160–1.345, *p* < 0.001). In multivariate logistic regression analysis, PSM length remained an independent prognostic factor for BCR (*p* < 0.001). **Conclusions**: Quantitative measurement of PSM length serves as an independent predictor of BCR following radical prostatectomy. We propose subclassifying R1 margins into R1a (<3.5 mm) and R1b (≥3.5 mm), which may enhance prognostic accuracy in pathological reporting.

## 1. Introduction

Prostate cancer (PCa) is the second most commonly diagnosed cancer among men and ranks as the fifth leading cause of cancer-related deaths worldwide, with approximately 375,000 deaths annually [1]. The lifetime risk of developing prostate cancer in men is estimated at 11.2% [2]. The incidence of prostate cancer increases with age and is rarely observed in individuals under the age of 40 [3].

The tumor-node-metastasis (TNM) classification system is used for staging prostate cancer. In cases of organ-confined (T1–T2) localized disease, treatment options include radical prostatectomy (RP), radiotherapy, hormone therapy, and ablative therapies. Among patients with localized or locally advanced prostate cancer and a life expectancy of more than 10 years, RP is the most commonly performed treatment [4]. The most frequently used parameters for assessing oncologic outcomes after RP are positive surgical margin (PSM) and biochemical recurrence (BCR) [5].

Among patients undergoing RP for clinically localized prostate cancer (LPCa), the incidence of positive surgical margins ranges from 6% to 22% [6]. Although PSM has been shown to be an independent risk factor for BCR, studies have found that not all patients with PSM share the same risk of BCR and that the length and extent of PSM may play a significant role in the risk of adverse oncological outcomes [6]. For instance, while some studies have reported that PSM lengths greater than 3 mm are associated with a higher risk of BCR [7,8,9,10,11], others have found no significant association between PSM extent and recurrence [12]. A recent meta-analysis suggested a hazard ratio of nearly 2.0 for a PSM ≥ 3 mm [13]. This variability highlights the need for better risk stratification among margin-positive patients to guide clinical decision-making. Furthermore, there is currently no clinical test that can reliably predict the likelihood of progression or biochemical recurrence in patients with positive margins following surgery. In clinical practice, patient monitoring is guided by the residual tumor (R) designation in the postoperative TNM pathology report, and patients are routinely followed with PSA testing every three months [6].

In this study, we aimed to investigate whether the quantitative length of the positive surgical margin in patients who underwent robot-assisted radical prostatectomy and were found to have a positive margin can serve as an early predictor of biochemical recurrence. Accordingly, we investigated the possibility of establishing a subgroup of R classification indicating PSM length in addition to R classification indicating PSM in the pathology reports of prostatectomy specimens.

## 2. Materials and Methods

Following the approval of the ethics committee on 17 September 2020 (Decision No. B.10.1.TKH.4.34.HGP.0.01/304), we retrospectively reviewed data from patients who underwent robot-assisted radical prostatectomy for prostate cancer in the Urology Department of our hospital between 30 July 2008 and 31 December 2019 and were found to have a positive surgical margin on pathological examination. This single-center study included 353 patients operated on by three experienced urologists using a standardized RARP technique. Pathological staging was conducted in accordance with the 2017 TNM classification. Prostatectomy specimens reported as R1 on pathology reports for positive surgical margins were re-evaluated by a uropathologist for surgical margins. The external surface of the prostate was marked with four-color ink (anterior–posterior; right–left) and H&E stained slides were re-analyzed. PSM was defined as cancerous tissue in contact with the inked surface and the total linear PSM length was measured in millimeters at all foci according to CAP guidelines [14]. Multifocal PSMs were summed for total length. The pathologist was not blinded, which is a potential source of bias.

Patients with negative surgical margins and those who had received neoadjuvant or adjuvant hormone therapy, preoperative radiotherapy, or adjuvant radiotherapy to the pelvic region were excluded from this study. Patients who received adjuvant or early salvage radiotherapy were also excluded to minimize confounding effects on BCR outcomes. Biochemical recurrence during the postoperative follow-up period was monitored via PSA testing. Follow-up involved PSA testing at 1, 3, 6, 9, 12, 18, and 24 months post-surgery and then annually. BCR was defined as a PSA level of ≥0.2 ng/mL in two consecutive samples. Follow-up ranged from 1 to 132 months (median 49.5 ± 33.4 months), with censoring for non-recurred patients at the last PSA. No patients were lost to follow-up, but some had shorter follow-ups due to this study ending.

Informed consent was waived due to the retrospective nature of this study, as approved by Institutional Review Board of the Health Sciences University Ümraniye Training and Research Hospital. All data were anonymized to protect patient privacy.

### Statistical Analysis

Data were analyzed using SPSS version 25.0 (SPSS^®^, Chicago, IL, USA). Percentages and frequencies were calculated for categorical variables, while mean, standard deviation, median, minimum, and maximum values were determined for continuous variables. The Shapiro–Wilk test was used to assess the normality of data distribution. Patients were grouped according to the presence [BCR (+)] or absence [BCR (−)] of biochemical recurrence. For comparisons between groups, the Student’s *t*-test was used for normally distributed data, and the Mann–Whitney U test was applied for non-normally distributed data. The Chi-square test was used for categorical data. Univariate regression analyses were performed to identify factors associated with biochemical recurrence. Cox proportional hazards regression assessed BCR predictors, including PSM length, preoperative PSA, clinical stage, tumor density, ISUP grade group, pathological stage, and lymph node dissection. Multicollinearity was evaluated using variance inflation factors (VIFs) in SPSS. The model’s discriminative ability was assessed using the C-statistic, calculated from risk scores derived from Cox regression. The sensitivity and specificity of PSM length for predicting BCR were assessed using ROC (Receiver Operating Characteristic) analysis, and the Youden index was used to determine the optimal cut-off value. ROC analysis was performed to compare the area under the curve (AUC) for the base model (without PSM length) and the full model (including PSM length), with statistical significance of the AUC difference tested. BCR (−) survival was analyzed using the Kaplan–Meier method, with statistical comparisons made using the Log-Rank test. The correlation between PSM length and time to BCR was assessed using Spearman correlation analysis. A *p*-value < 0.05 was considered statistically significant.

## 3. Results

A total of 353 patients who underwent robot-assisted radical prostatectomy in our clinic and were found to have positive surgical margins on pathology were included in this study. The patients’ demographic characteristics, preoperative findings, and postoperative outcomes were analyzed, and comparisons were made between those who developed BCR and those who did not.

Biochemical recurrence was detected in 96 patients (27.1%). The mean age of the patients was 62.7 ± 6.6 years, and the median follow-up period was 49.5 ± 33.4 months. Preoperative and perioperative findings are presented in Table 1.

The mean tumor volume was 10.2 ± 10.5 cc in the BCR (+) group and 6.8 ± 6.9 cc in the BCR (−) group (*p* = 0.001). Tumor density was measured as 0.18 ± 0.17 cc/cc in the BCR (+) group and 0.13 ± 0.12 cc/cc in the BCR (−) group (*p* = 0.002).

Regarding clinical staging, among the 282 patients with clinical stage 1 disease, 216 (76.6%) were in the BCR (−) group, and 66 (23.4%) were in the BCR (+) group. Among the 66 patients with clinical stage 2 (determined by positive digital rectal examination), 39 (59.1%) were in the BCR (−) group, and 27 (40.9%) in the BCR (+) group. Of the five patients with clinical stage 3 disease, two (40%) were in the BCR (−) group, and three (60%) were in the BCR (+) group (*p* = 0.004).

A statistically significant difference was observed between the two groups regarding ISUP and pathological staging. Pathological lymph node positivity was found in 61.5% of the BCR (+) group and 38.5% of the BCR (−) group (*p* = 0.005). Regarding positive surgical margins, PSMs were evaluated across five anatomical locations: posterior, apex, bladder neck, anterior, and multiple sites. No statistically significant difference was found between the two groups regarding PSM location (*p* = 0.3).

**Figure 1:**
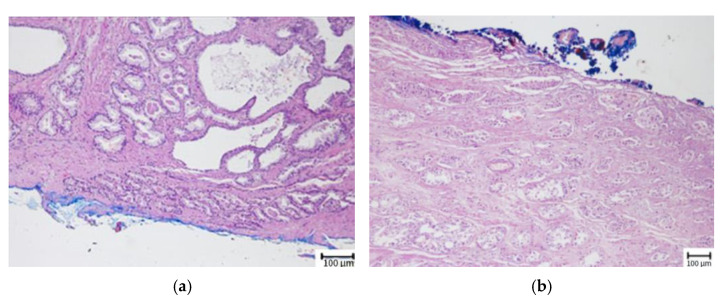
Determination of the length of the surgical margin in the area stained with ink in the pathology specimen. (**a**) Tumor cells touching the surgical margin ink in a focal area; PCS length < 3.5 mm (H&E, ×10). (**b**) Tumor cells continuing in a wide area along the surgical margin ink; PCS length ≥ 3.5 mm (H&E, ×10).

When the PSM length was examined, it was found to be 6.6 ± 4.8 mm in the BCR (+) group and 3.1 ± 3.2 mm in the BCR (−) group, and a statistically significant difference was identified between the two groups (*p* < 0.001). In our study, using the Youden index, the cut-off value for PSM length predicting biochemical recurrence was determined as 3.5 mm (Figure 1). Among the 353 patients, 215 had a surgical margin length <3.5 mm, of whom 21 (9.8%) developed BCR. Among the 138 patients with a surgical margin length ≥3.5 mm, BCR occurred in 75 (54.3%). Based on these findings, the likelihood of BCR was found to be statistically significantly higher in patients with a surgical margin length ≥3.5 mm (*p* < 0.001).

No statistically significant difference was found between the groups regarding postoperative complication rates according to the Clavien–Dindo Classification (*p* = 0.63).

In terms of predicting biochemical recurrence, univariate analysis revealed statistically significant associations with preoperative PSA level, clinical stage, specimen tumor density, specimen ISUP grade group, pathological tumor stage, pathological lymph node (pN) positivity, and the length of the positive surgical margin (*p* = 0.02, *p* = 0.004, *p* = 0.003, *p* = 0.017, *p* = 0.018, *p* = 0.009, and *p* < 0.001, respectively).

In multivariable Cox regression analysis, PSM length remained a significant predictor. Cox proportional hazards regression identified PSM length as an independent predictor of biochemical recurrence (HR: 1.113, 95% CI: 1.076–1.152, *p* < 0.001) and pN positivity as a trend (HR: 1.282, 95% CI: 0.994–1.654, *p* = 0.056) (Table 2).

**Figure 2:**
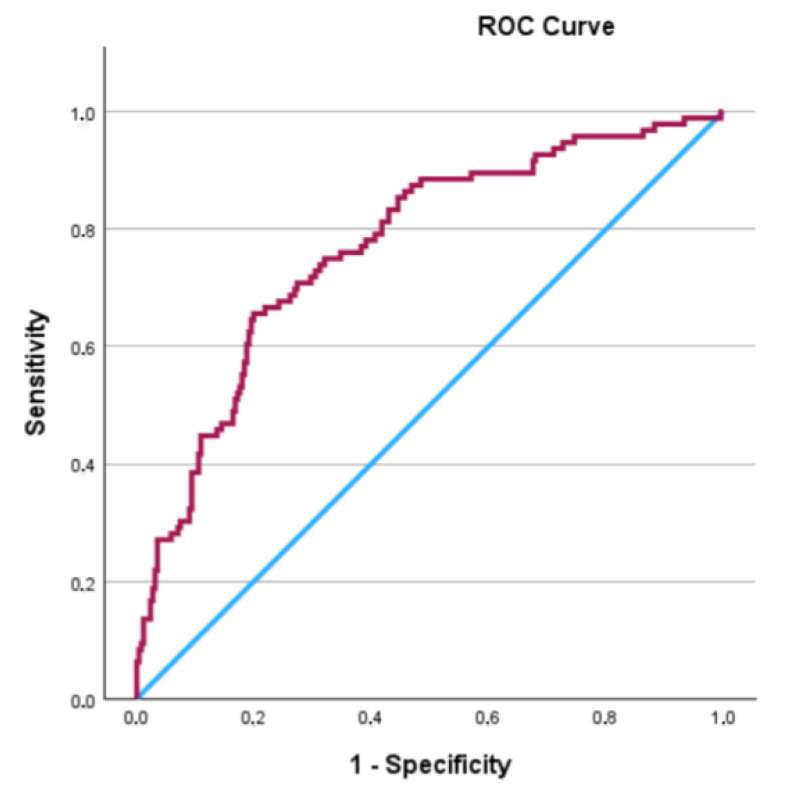
ROC curve of the Cox regression model for predicting BCR (AUC = 0.770).

To assess the predictive ability of positive surgical margin length for biochemical recurrence, ROC analysis was performed. Based on the ROC analysis of PSM length, the AUC of the Cox regression model was 0.770 (95% CI: 0.714–0.826, *p* < 0.001), indicating good discriminatory ability for predicting BCR. The model achieved a sensitivity of 0.656 and a specificity of 0.801 (*p* < 0.001) (Figure 2).

**Figure 3:**
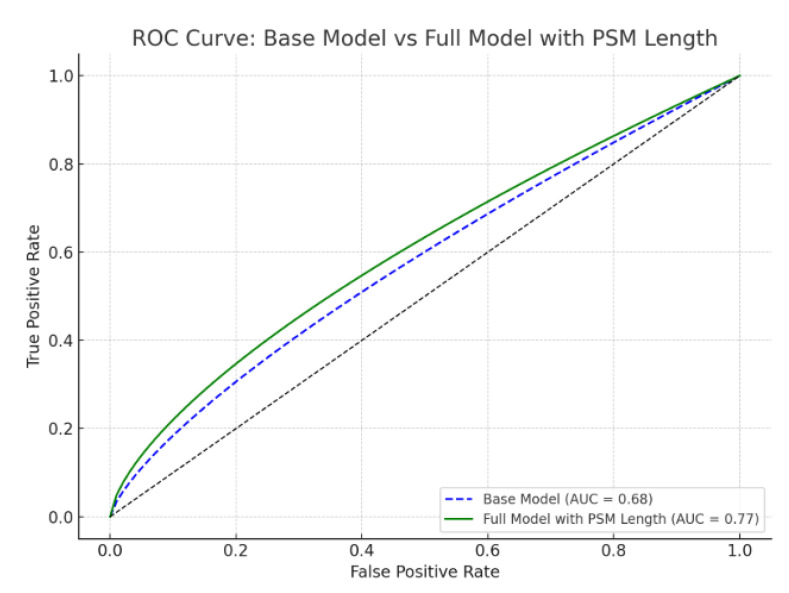
ROC curves: impact of PSM length on BCR prediction.

When PSM length was added to a baseline model including preoperative PSA, clinical stage, tumor density, ISUP grade group, pathological stage, and pN positivity, the AUC improved from 68% to 77%, highlighting the prognostic value of PSM length. ROC analysis demonstrated that the base model had an AUC of 0.68, which improved to 0.77 with the inclusion of PSM length (*p* = 0.03), indicating enhanced predictive ability (Figure 3).

The model’s C-statistic was 0.78, indicating good discrimination. Multicollinearity assessment using variance inflation factors (VIFs) showed values ranging from 1.149 to 1.304 (all < 5), suggesting no significant intercorrelation among variables.

**Figure 4:**
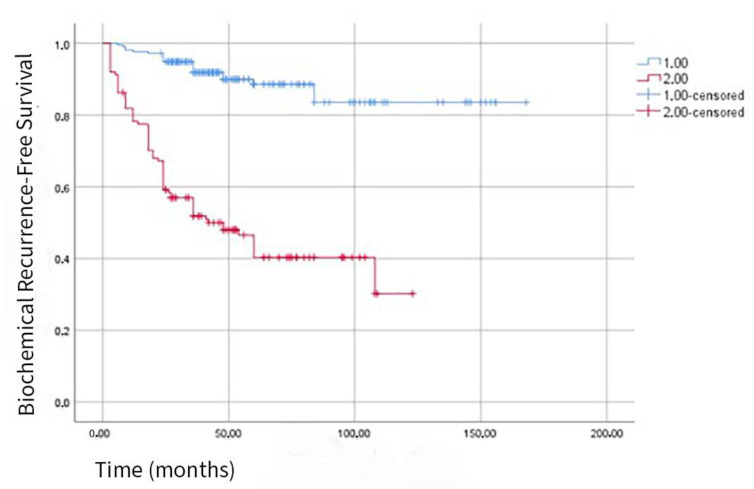
Kaplan–Meier curve for biochemical recurrence-free survival. Comparison of patients with positive surgical margin (PSM) length < 3.5 mm and ≥3.5 mm (Group 1.00 → PSM < 3.5 mm; Group 2.00 → PSM ≥ 3.5 mm) (Log Rank *p*: <0.001).

According to the Kaplan–Meier survival curve, 5-year biochemical recurrence-free (BCR−) survival was 87% in patients with PSM < 3.5 mm and 40% in patients with PSM ≥ 3.5 mm, indicating a statistically significant association between PSM length and BCR (−) survival (*p* < 0.001) (Figure 4).

The mean survival time was calculated as 148 ± 4.45 months (95% CI: 139.51–156.97) in the group with PSM < 3.5 mm and 62 ± 4.6 months (95% CI: 52.96–71.24) in the group with PSM ≥ 3.5 mm.

**Figure 5:**
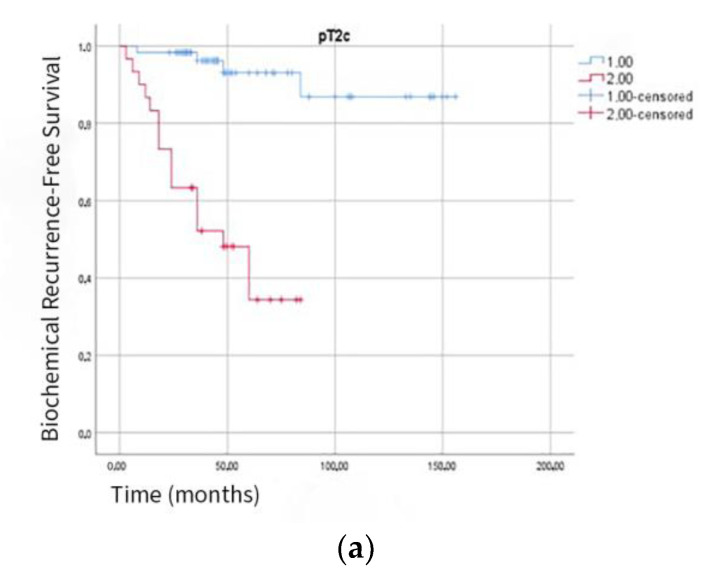

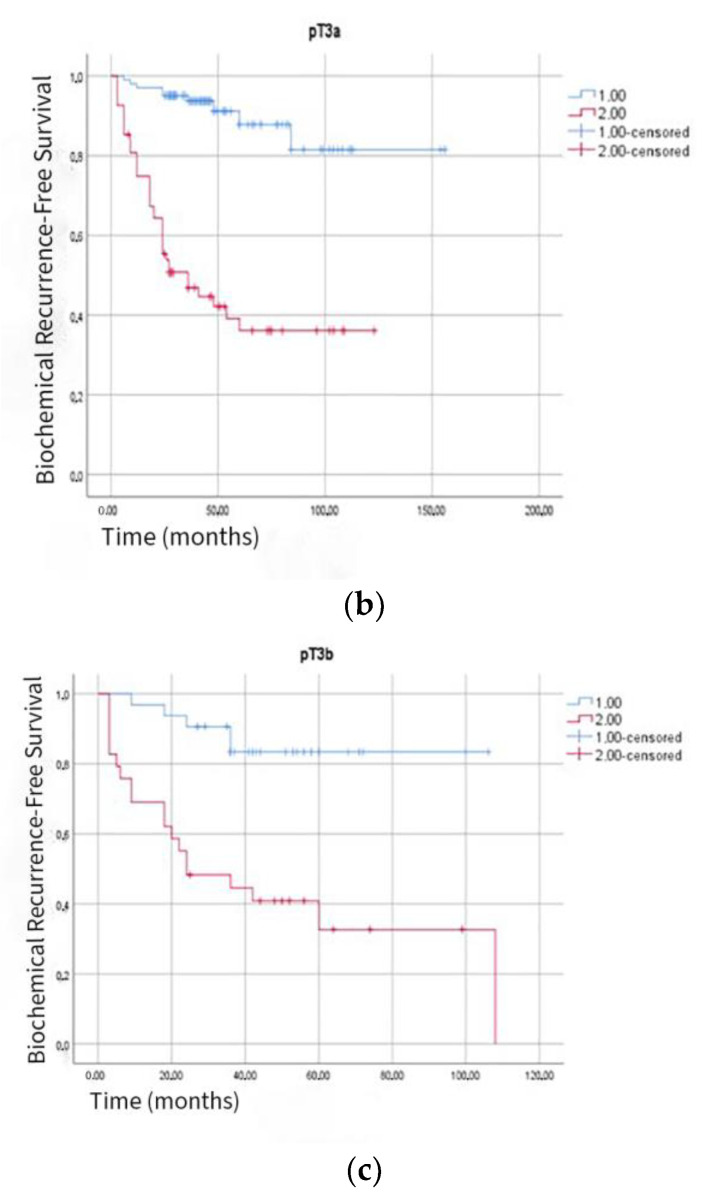
(**a**). Kaplan–Meier curve for biochemical recurrence-free survival. Comparison of patients with pathological stage T2c and positive surgical margin (PSM) length and ≥3.5 mm (Group 1.00 → PSM < 3.5 mm; Group 2.00 → PSM ≥ 3.5 mm) (Log Rank *p*: <0.001). (**b**). Kaplan–Meier curve for biochemical recurrence-free survival. Comparison of patients with pathological stage T3a and positive surgical margin (PSM) length and ≥3.5 mm (Group 1.00 → PSM < 3.5 mm; Group 2.00 → PSM ≥ 3.5 mm) (Log Rank *p*: <0.001). (**c**). Kaplan–Meier curve for biochemical recurrence-free survival. Comparison of patients with pathological stage T3b and positive surgical margin (PSM) length and ≥3.5 mm (Group 1.00 → PSM < 3.5 mm; Group 2.00 → PSM ≥ 3.5 mm) (Log Rank *p*: <0.001).

Furthermore, the impact of PSM ≥ 3.5 mm on BCR (−) survival was considerably greater, regardless of the subgroup. This significant relationship was also observed in patients classified as pT2c, pT3a, or pT3b (*p* < 0.001). Among patients with pathological stage T2c (pT2c), the BCR (−) survival rate was 93.2% in the PSM < 3.5 mm group versus 43.3% in the PSM ≥ 3.5 mm group (Figure 5a). In patients with pathological stage T3a (pT3a), the BCR (−) survival was 91.2% in the PSM < 3.5 mm group and 42.6% in the PSM ≥ 3.5 mm group (Figure 5b). In patients with pathological stage T3b (pT3b), the BCR (−) survival was 84.24% in the PSM < 3.5 mm group and 34.5% in the PSM ≥ 3.5 mm group (Figure 5c).

## 4. Discussion

Positive surgical margin is a frequently encountered condition following radical prostatectomy. The incidence of PSM depends on various factors, including tumor biology, patient characteristics, pathological assessment methods, and surgical techniques [15].

In our cohort of 1966 RARPs, the PSM rate was 18% (353/1966), with BCR in 27.1% of cases (96/353). Prior studies report PSM rates of 16–25% for open RP, 11–30% for laparoscopic RP, and 12–25% for RARP [16], aligning with our findings.

Preoperative PSA, D’Amico risk classification, pathological T stage, pathological Gleason score or ISUP grade group, pathological lymph node positivity, and the presence of PSM are important factors for predicting the likelihood of BCR [17,18,19]. In our study, parameters predicting biochemical recurrence after RP were identified as preoperative PSA level, clinical stage, specimen tumor density, specimen ISUP grade group, pathological tumor stage, pN positivity, and length of the positive surgical margin. Univariate analysis identified preoperative PSA, clinical stage, tumor density, ISUP grade group, pathological stage, lymph node positivity, and PSM length as BCR predictors. However, only PSM length remained significant in Cox regression (HR: 1.113, *p* < 0.001), with lymph node dissection showing a trend (HR: 1.282, *p* = 0.056).

Many studies have reported that pathological lymph node positivity increases the risk of BCR. In a study by Schiavina et al., the extent of pelvic lymph node dissection was found to be associated with biochemical recurrence rates in patients with intermediate- and high-risk prostate cancer [20]. However, in our study, no significant association was found, even if close, in the multivariate Cox regression analysis (*p* = 0.56). We believe this may be due to the fact that not all patients underwent extended lymph node dissection.

The literature review regarding pathological tumor stage revealed that many studies identified it as an independent predictive factor for BCR development in both univariate and multivariate analyses. In an analysis of a large cohort of 498 patients with PSM, PSM was shown to be an independent predictor of BCR in patients classified as pT2 and pT3a. However, this association was not found to be significant in patients classified as pT3b and pT4 [21]. Therefore, in patients with lymph node or seminal vesicle invasion, prognosis appears to be determined more by the presence of micrometastases responsible for systemic disease spread rather than PSM changes [22]. In our study, we observed an increased risk of BCR with advancing pathological stage. But it was not significant in multivariable Cox regression analysis. This result may be due to the relatively short follow-up period (median of 49.5 months), which may have missed late recurrences. Moreover, we observed that PSM length significantly increased the risk of BCR not only in the pT2 and pT3a groups but also in the pT3b group.

Several studies have concluded that PSM is an independent factor for BCR in patients with PCa after RP. Swindle et al. reported that positive surgical margins were a significant predictor of recurrence after PCa treatment [15]. Ploussard et al. found that positive surgical margins were a predictor of PSA recurrence [21]. Based on these studies in the literature, it can be stated that positive surgical margins are an indicator of poor prognosis in PCa.

Although PSM has been shown to be an independent risk factor for BCR, studies have found that not all patients with PSM share the same risk of BCR and that the length and extent of PSM may play a significant role in the risk of adverse oncological outcomes [23,24]. This suggests that evaluating PSM in isolation is not sufficient to predict the likelihood of BCR. Therefore, a need has arisen to further specify the surgical margin, and it has been hypothesized that tumor extent at the surgical margin (PSM length and PSM location) may be associated with BCR [23,24].

When we look at studies evaluating the relationship between PSM location and BCR, a study by Choo et al. reported that PSMs observed at the apex and bladder neck increased the likelihood of BCR [25]. Sooriakumaran et al. stated that the highest risk of BCR was associated with apical PSMs, followed by anterior PSMs [26]. In contrast, the latest edition of the AJCC states that the location of the PSM is not important for BCR [27]. In conclusion, the literature presents varying results regarding which PSM locations should be considered. Similarly, our study found no significant relationship between PSM location and the risk of BCR, in line with the AJCC.

When reviewing studies examining the relationship between PSM length and BCR, a study by Ochiai et al. showed similar rates of biochemical recurrence-free survival in cases with PSM lengths of <1 mm and 1–3 mm, whereas a PSM length > 3 mm was associated with an increased incidence of biochemical recurrence [7]. Sooriakumaran et al. analyzed a large series of 189 patients with at least five years of follow-up. They reported that patients with a PSM > 3 mm had a significantly higher risk of BCR [8]. The same result was found in the study by Udo et al., which indicated that a PSM length > 3 mm increased the likelihood of BCR [9]. Similarly, Cao et al. reported that an increase in linear PSM length was an independent predictive factor for BCR [10]. Likewise, Dev et al. analyzed a cohort of approximately 4000 patients who underwent RP and found that a PSM length > 3 mm was associated with BCR [11]. Conversely, a study by Leite et al. concluded that the extent of PSM was not related to BCR [12].

The findings of our study are supported by this literature regarding the role of positive surgical margin (PSM) length in biochemical recurrence (BCR). Previous studies investigating the relationship between PSM length and BCR have commonly adopted a 3 mm threshold to define “extensive” margins, with worse outcomes reported above this cutoff [7,8,11]. Notably, a recent systematic review and meta-analysis by John et al. (Prostate Cancer Prostatic Dis. 2023) concluded that longer PSM length was independently associated with an elevated risk of BCR, reporting a hazard ratio (HR) of 1.99 for PSM ≥ 3 mm compared to <3 mm [13]. This meta-analysis’s HR of approximately 2.0 lends support to our observed HR of 1.113, although the smaller sample size in our study (96 events) limits statistical power for multiple predictors. In contrast to the commonly cited 3 mm threshold, our study identified a 3.5 mm cutoff using ROC analysis and the Youden index, which closely aligns with the established threshold but reflects the specific characteristics of our cohort and measurement approach. This minor discrepancy in cutoff values may be attributed to variations in patient populations, surgical techniques, or pathological assessment methods. Multicollinearity diagnostics (VIFs ranging from 1.149 to 1.304, calculated using SPSS) and a C-statistic of 0.78 validate the robustness of our model. The ROC analysis demonstrated that the inclusion of PSM length improved the area under the curve (AUC) from 0.68 for the base model to 0.77 for the full model (*p* = 0.03), underscoring its utility as an independent predictor. Our findings contribute to the growing body of evidence establishing PSM length as a critical prognostic factor and support the subclassification of R1 margins into R1a (<3.5 mm) and R1b (≥3.5 mm). This approach aligns with the 2019 College of American Pathologists (CAP) recommendation for reporting total linear PSM length, potentially enhancing the prognostic utility of pathology reports [14].

When we review studies investigating the relationship between PSM length and BCR (−) survival, in a 2013 study by Sooriakumaran et al., five-year BCR (−) survival was 81% in patients with PSM < 3 mm and 65% in those with PSM ≥ 3 mm [8]. Likewise, in a 2015 study by Lee et al., five-year BCR (−) survival was 83% in patients with PSM < 3 mm and 54% in those with PSM ≥ 3 mm [28]. Our ROC-derived 3.5 mm cutoff (AUC 0.77) offers a modest improvement in predictive performance, with 5-year BCR-free survival rates of 87% versus 40% (*p* < 0.001). This outperforms prior reports, which noted 5-year BCR-free survival rates of 81% versus 65% for PSM < 3 mm versus ≥3 mm [8] and 83% versus 54% [28]. This demonstrates a significant relationship between PSM length and BCR (−) survival. Furthermore, the impact of PSM ≥ 3.5 mm on BCR (−) survival was considerably greater, regardless of the subgroup. This significant relationship was also observed in patients classified as pT2c, pT3a, or pT3b. Based on these results, we suggest that PSM length should be considered alongside other prognostic factors when monitoring patients with positive surgical margins, as PSM length has been identified as an independent prognostic factor in predicting biochemical recurrence.

The proposed subclassification of R1 margins into R1a (<3.5 mm) and R1b (≥3.5 mm) has practical implications for clinical management. Patients with R1b margins (≥3.5 mm) exhibit a significantly higher risk of BCR (54.3% vs. 9.8% for R1a), suggesting they may benefit from closer monitoring, such as more frequent PSA testing, or consideration of early adjuvant therapies like radiotherapy, particularly if other adverse features are present. Conversely, patients with R1a margins (<3.5 mm) could potentially be managed more conservatively if other prognostic factors are favorable, reducing overtreatment. Incorporating this subclassification into routine pathology reports could enhance prognostic accuracy and guide personalized treatment decisions, although further validation in larger, multicenter cohorts is needed to establish its broader clinical utility.

This study has several limitations. First, the median follow-up period of 49.5 months is relatively short to medium-term, which may not capture late biochemical recurrences, as BCR in prostate cancer can occur even beyond five years after surgery. Some patients who are currently BCR-free might develop recurrence later, potentially underestimating the true recurrence rate. Second, we lack data on clinical recurrence, metastasis, or prostate cancer-specific survival, which limits our ability to assess the broader prognostic implications of PSM length. Third, this is a single-center study involving only robot-assisted radical prostatectomies (RARP) performed by a small group of experienced urologists, which may limit the generalizability of our findings to other surgical approaches (e.g., open or laparoscopic RP) or practice settings. While PSM length should theoretically have a similar prognostic impact regardless of surgical technique, this requires validation in more diverse cohorts. Fourth, the exclusion of patients who received neoadjuvant or adjuvant hormone therapy may have introduced selection bias, as these patients likely had more adverse features (e.g., extensive margins; higher stage) and were treated with immediate systemic therapy. Similarly, patients receiving adjuvant or early salvage radiotherapy were excluded to minimize confounding effects on BCR outcomes. Consequently, our cohort may represent a more favorable subset of margin-positive patients.

## 5. Conclusions

The quantitative length of the positive surgical margin in pathological evaluations after radical prostatectomy has been identified as an independent prognostic factor in predicting biochemical recurrence. We believe that subclassifying positive surgical margins in pathology reports of radical prostatectomy specimens, not just as R1, but as R1a: <3.5 mm and R1b: ≥3.5 mm, would provide more informative prognostic data. External validation with a large number of patients and a longer follow-up is necessary to evaluate this classification.

## Figures and Tables

**Table 1:** Postoperative pathological, oncological, and functional findings of patients with positive surgical margins.

Variable	All Patients (n = 353)	BCR (−) (n = 257)	BCR (+) (n = 96)	*p*-Value
Prostate volume (mean ± SD, cc)	54.4 ± 19.7	54.4 ± 20.2	54.2 ± 18.5	0.936
Tumor volume (mean ± SD, cc)	7.7 ± 8.2	6.8 ± 6.9	10.2 ± 10.5	0.001
Tumor density (mean ± SD, cc/cc)	0.14 ± 0.14	0.13 ± 0.12	0.18 ± 0.17	0.002
ISUP Grade Group:				<0.001
1 (Gleason ≤ 6)	43	29 (67.4%)	14 (32.6%)	
2 (Gleason 3 + 4 = 7)	165	134 (81.2%)	31 (18.8%)	
3 (Gleason 4 + 3 = 7)	101	73 (72.3%)	28 (27.7%)	
4 (Gleason 8)	26	15 (57.7%)	11 (42.3%)	
5 (Gleason ≥ 9)	18	6 (33.3%)	12 (66.7%)	
Lymph Node Dissection:				0.005
pNx, pN0	340	252 (74.1%)	88 (25.9%)	
pN1	13	5 (38.5%)	8 (61.5%)	
Clavien Complication Score:				0.638
1	289	208 (72%)	81 (28%)	
2	52	39 (75%)	13 (25%)	
3	12	10 (83.3%)	2 (16.7%)	
Pathological Stage:				0.02
T2a	20	19 (95%)	1 (5%)	
T2b	13	11 (84.6%)	2 (15.4%)	
T2c	89	68 (76.4%)	21 (23.6%)	
T3a	170	122 (71.8%)	48 (28.2%)	
T3b	61	37 (60.7%)	24 (39.3%)	
PSM Location:				0.3
Posterior	162	116 (71.6%)	46 (28.4%)	
Apex	69	53 (76.8%)	16 (23.2%)	
Bladder neck	27	20 (74.1%)	7 (25.9%)	
Anterior	28	24 (85.7%)	4 (14.3%)	
Multiple	67	44 (65.7%)	23 (34.3%)	
PSM Length (mm):	4.1 ± 4.03	3.1 ± 3.2	6.6 ± 4.8	<0.001
<3.5 mm	215	194 (75.5%)	21 (21.9%)	
≥3.5 mm	138	63 (24.5%)	75 (78.1%)	

**Table 2:** Multivariable Cox regression analysis for predicting biochemical recurrence.

Factors	HR	95% CI	*p*
Pre-biopsy PSA	1.016	0.995–1.038	0.142
Clinical Stage	1.333	0.873–2.036	0.183
Specimen Tumor Density	2.047	0.484–8.661	0.33
Specimen ISUP Grade Group	1.228	0.987–1.527	0.065
Pathological Tumor Stage	1.123	0.862–1.463	0.391
pN Positivity	1.282	0.994–1.654	0.056
PSM Length	1.113	1.076–1.152	<0.001

## Data Availability

The data presented in this study are available on request from the corresponding author. The data are not publicly available due to privacy restrictions.

## Abbreviations

The following abbreviations are used in this manuscript:

BCR	Biochemical Recurrence
CI	Confidence Interval
H&E	Hematoxylin and Eosin
HR	Hazard Ratio
PSA	Prostate-Specific Antigen
PSM	Positive Surgical Margin
ROC	Receiver Operating Characteristic
RP	Radical Prostatectomy
RARP	Robot-Assisted Radical Prostatectomy
SD	Standard Deviation
